# Bile Acids: Key Regulators and Novel Treatment Targets for Type 2 Diabetes

**DOI:** 10.1155/2020/6138438

**Published:** 2020-07-17

**Authors:** Yingjie Wu, An Zhou, Li Tang, Yuanyuan Lei, Bo Tang, Linjing Zhang

**Affiliations:** ^1^Department of Gastroenterology, Xinqiao Hospital, Third Military Medical University, Chongqing 400037, China; ^2^Zhongshan School of Medicine, Sun Yat-sen University, Guangzhou 510030, China; ^3^Department of Nuclear Medicine, Southwest Hospital, Third Military Medical University, Chongqing 400038, China

## Abstract

Type 2 diabetes mellitus (T2DM), characterized by insulin resistance and unclear pathogenesis, is a serious menace to human health. Bile acids are the end products of cholesterol catabolism and play an important role in maintaining cholesterol homeostasis. Furthermore, increasing studies suggest that bile acids may regulate glucose tolerance, insulin sensitivity, and energy metabolism, suggesting that bile acids may represent a potential therapeutic target for T2DM. This study summarizes the metabolism of bile acids and, more importantly, changes in their concentrations, constitution, and receptors in diabetes. Furthermore, we provide an overview of the mechanisms underlying the role of bile acids in glucose and lipid metabolism, as well as the occurrence and development of T2DM. Bile acid-targeted therapy may represent a valid approach for T2DM treatment.

## 1. Introduction

T2DM is generally considered a progressive, incurable, and increasingly prevalent illness characterized by insulin resistance and a deficiency in the absence of autoimmune beta-cell destruction, accounting for 90-95% of all diabetes cases [1]. Furthermore, it has become the most challenging endocrine disease and a leading cause of mortality worldwide, which by all predictions will only increase [2]. With rapid urbanization, economic growth, and changes in lifestyles, the prevalence of T2DM in China is increasing significantly, representing a serious problem that causes a significant burden on society [3]. The pathogenesis of T2DM is not yet entirely clear, and some evidence suggests it may be related to obesity, ethnicity, and environmental risk factors [4]. Recently, emerging evidence has suggested that insulin resistance may be the most important contributor [5]. Obese individuals tend to develop inflammation in their fat tissue, reducing the sensitivity of fat cells to insulin and inducing the development of T2DM and diminished effects of insulin [1]. Furthermore, T2DM frequently causes microvascular pathological changes, leading to stroke, heart failure, renal failure, and myocardial infarction in late stages, leading to poor prognosis and quality of life in patients [6]. Currently, treatment of T2DM comprises five primary methods: education for patients, self-monitoring of blood glucose, diet, exercise, and medication [7]. However, these methods are expensive and inefficient. Hence, diagnosis is often delayed until complications have arisen and financial costs for the treatment of T2DM have become significant, indicating the urgent need to develop new and efficient therapies and prevention methods for the control of type 2 diabetes.

Current studies have shown that gut hormones, such as ghrelin, play an important role in mediating feeding, which is significant to the development of T2DM [8]. As the endogenous ligand for the growth hormone secretagogue receptor (GHS-R) [9], ghrelin not only induces growth hormone release but also enhances food intake and stimulates adiposity [10, 11]. Primarily synthesized by X/A-like cells in the gastric oxyntic glands [9], acyl ghrelin releases NPY and AgRP by activating tGHS-Rs to stimulate food intake, body weight gain, and diabetic hyperphagia [12]. Additionally, gut hormones such as ghrelin have a vital influence on glucose metabolism [13] and T2DM remission; one study revealed that mice lacking acyl ghrelin demonstrated reduced fasting blood glucose levels and improved insulin sensitivity for controlling feeding blood glucose [14]. Moreover, other gut hormones, such as glucose-dependent insulinotropic polypeptide and glucagon-like peptide 1, hold the same promising potential in T2DM research [8].

Bile acids are the end products of cholesterol catabolism and play an important role in maintaining cholesterol homeostasis and preventing the buildup of toxic metabolites, as well as the accumulation of cholesterol [15]. Studies have demonstrated that bile acids are closely associated with the intestinal microbiota, which intimately affects gut hormones [16]. Regulation of feeding, metabolism, disease development, and homeostasis may be the result of their interactions and mutual influence [17]. On the one hand, bile acids not only facilitate transport of lipids and intestinal absorption but are also inflammatory agents and signaling molecules that effectively activate cell signaling pathways that regulate glucose, lipids, and energy metabolism [18]. On the other hand, accumulating studies have suggested that bile acids could activate certain receptors, such as the farnesoid X receptor (FXR) and the transmembrane G protein-coupled receptor 5 (TGR5), which improves glucose tolerance, insulin sensitivity, and energy metabolism [19]. The effects of these receptors suggest that bile acids may represent a potential therapeutic target for treating T2DM. This review is aimed at summarizing the effects of and changes in bile acids and their main receptors, such as FXR and TGR5, in T2DM development and their promise of representing potential treatment targets for T2DM.

## 2. Metabolism of Bile Acids

Bile acids (BAs) are significant bile components synthesized in the liver by cholesterol, secreted into the bile duct, and concentrated in the gallbladder, and they serve as amphipathic biological detergents for lipid metabolism [20]. Most bile acids are reabsorbed and recycled *via* enterohepatic circulation, and approximately 5% are lost in feces or serve as substrates for biotransformation and metabolism in the intestinal microbiota [20]. These bacteria are responsible for the dissociation of bile acids from glycine or taurine mediated by hydrolytic enzymes and hydroxyl oxidation [20].

**Figure 1:**
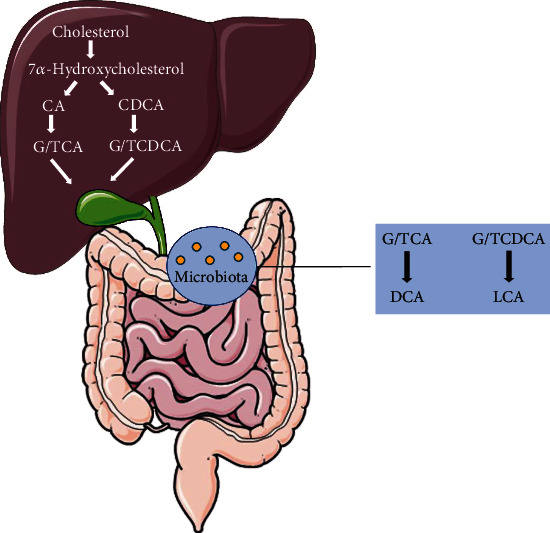
Bile acid synthesis and metabolism. Schematic representation of synthetic pathways of primary bile acids in hepatocytes and secondary bile acids in the intestine.

The primary bile acids chenodeoxycholic acid (CDCA) and cholic acid (CA) are synthesized *via* two pathways utilizing approximately fifteen enzymes from cholesterol in the liver [20]. The rate-limiting enzyme, cytochrome P450 cholesterol 7*α*-hydroxylase (CYP7A1), triggers the classical pathway, converting cholesterol into 7*α*-hydroxycholesterol and producing most of the BA pool [21]. CA is formed through subsequent modification by a series of enzyme cascades (CYP8B1, AKR1D1, AKR1C4, and CYP27A1), while CDCA is synthesized by the same enzyme cascades except for CYP8B1 [21]. The rest of the BA pool is synthesized by cytochrome P450 27*α*-hydroxylase (CYP27A1) *via* an alternative pathway. First, cholesterol may be oxidized to 27-hydroxycholesterol with the help of CYP7B1. Then, 27-hydroxycholesterol is transformed into CDCA by CYP7B1 [21] (Figure 1). The dissociative BAs mentioned above transform into conjugated BAs after their conjugation to either glycine (primarily in humans) or taurine (predominantly in mice) by bile acid-amino acid transferase (BAT) and bile acid-CoA synthase (BACS) enzymes. Next, conjugated BAs are secreted into the bile canaliculi *via* the bile salt export pump and BA transporters MRP2 and MDR1A [22]. Subsequently, they accumulate and are stored and concentrated in the gallbladder. When cholecystokinin (CCK) is secreted by enteroendocrine I cells, the gallbladder is stimulated to contract and secrete bile into the duodenum to participate in the digestion and solubilization of ingested lipids. Approximately 95% of intestinal BAs are reabsorbed by enterocytes from the distal ileum through the apical sodium-dependent BA transporter (ASBT/SLC10A2) or the ileal bile acid transporter (IBAT) [23, 24]. These absorbed BAs are cleared by active transporters in the sinusoidal membrane of hepatocytes (NTCP, OAT, OATP, and mEH) when they return to the liver through the superior mesenteric and portal veins [25]. These redissociated BAs return to hepatocytes, along with newly formed bile acids, and are then secreted into the bile ducts, a process known as enterohepatic circulation. A portion of reabsorbed BAs will successfully escape hepatic recapture and reach the peripheral tissues *via* systemic circulation, performing signaling functions on several peripherally expressed BA receptors [21, 26]. The remaining 5% of BAs are excreted in the feces or serve as substrates for biotransformation, where they will be converted into secondary bile acids by the intestinal microbiota: deoxycholic acid (DCA) is formed from CA, lithocholic acid (LCA) is formed from CDCA, and ursodeoxycholic acid (UDCA) is formed in humans *via* 7*α*-dehydroxylation [24, 27].

## 3. Bile Acid Metabolism Alterations in T2DM

Recently, accumulating studies have shown that bile acids are involved in systemic metabolism, pancreatic islet insulin resistance, hyperglycemia regulation, and energy expenditure [28, 29]. Abnormal BA metabolism is closely related to a variety of metabolic diseases, such as obesity, dyslipidemia, and nonalcoholic fatty liver disease. In addition, bile acids have been proven to be involved in glucose and lipid metabolism [22]. Therefore, we will focus on changes in BA metabolism in T2DM.

### 3.1. Changes in Total Bile Acids in T2DM

Some studies have shown that during the feeding state, total BA concentration in the setting of T2DM is significantly elevated compared to nondiabetic controls [30–32]. Furthermore, elevated total bile acid concentrations were positively correlated with increasing meal fat content [30]. For T2DM patients, total bile acid levels were positively correlated with triglycerides, insulin resistance index, blood pressure, and BMI, suggesting a relationship between total BA content and T2DM [33]. These findings suggest that total BA concentrations tend to increase in the setting of diabetes. Although the repeatability and authenticity of these experiments need to be further verified and the mechanism and causes of increased total BA concentration are not fully understood, we speculate that increased levels of total bile acids might be either a manifestation or cause of T2DM or may represent a link in the causal chain.

### 3.2. Changes in Bile Acid Composition in T2DM

Changes in bile acid composition have been verified in both clinical trials and animal models of type 2 diabetes mellitus. Increased concentrations of deoxycholic acid and decreased concentrations of chenodeoxycholic acid were observed in T2DM patients [34–38]. In T2DM patients, CDCA, DCA, and CA were also significantly increased, and CDCA, CA, and to a lesser extent DCA were positively associated with insulin resistance. Another study demonstrated that compared to nondiabetic controls, glyco-BAs are elevated in T2DM [39]. Moreover, glucose and insulin can boost histone acetylation of CYP7A1 chromatin, leading to stimulation of CYP7A1. CYP7A1 stimulation then activates the classic BA synthesis pathway and increases serum bile acids, leading to higher proportions of CA and DCA to CDCA and suggesting that abnormal glucose metabolism may affect bile acid metabolism in T2DM [40, 41]. In mouse models, inhibition of CA synthesis improves glucose homeostasis and prevents diet-induced obesity. However, increased levels of CA may contribute to dyslipidemia, diabetes, and obesity by stimulating cholesterol absorption [40–42].

Clearly, changes in the concentration and composition of the BA pool should not be ignored in T2DM. Although a few studies have been performed, evidence of these changes is still under investigation. Understanding changes in the bile acid pool is of great significance to the pathogenesis of T2DM.

## 4. Bile Acids in the Regulation of Glucose Homeostasis

**Figure 2:**
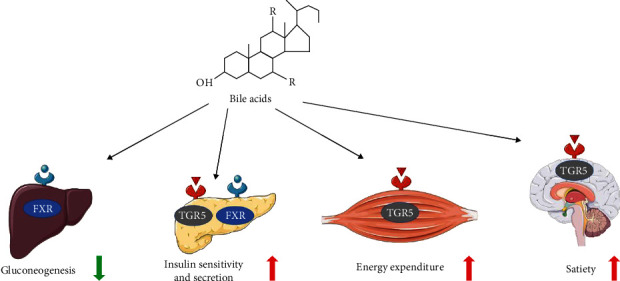
Bile acids in regulation of glucose homeostasis.

Currently, with far more understanding of the regulation of bile acid metabolism, we now know that it is an important pathway in glucose metabolism [20] (Figure 2). Bile acid metabolism is regulated by precise feedback mechanisms from two receptors called FXR and TGR5, and the differential affinity of the bile acids towards FXR and TGR5 was summarized in Table 1.

### 4.1. Regulation of Glucose Homeostasis and Bile Acid Metabolism by FXR

The nuclear receptor farnesoid X receptor (FXR), a key regulator of glucose metabolism, is significant not only to bile acid metabolism in the liver but also to biliary BA secretion and intestinal BA absorption [42–60]. FXR is easily activated by both free and conjugated bile acids due to its high expression levels in the liver. Activation of FXR in the liver subsequently increases the excretion of bile acids into the intestine and inhibits activity of CYP7A1, the rate-limiting enzyme of the classic pathway mentioned above, by increasing transcription of the inhibitory small heterodimer partner (SHP) [61]. FXR activation prevents the accumulation of bile acids in the liver *via* transcriptional induction of apical transporters, such as BSEP and MRP2 [62, 63]. To evaluate the function of FXR in regulating BA homeostasis and detoxification in response to bile duct ligation (BDL), many experiments have been performed in FXR knockout mice. These mice exhibited enhanced CYP7A1 mRNA expression and increased BA pools [64, 65]. Accompanied by a lack of CYP7A1 inhibition, FXR null mice presented with more serious hepatotoxicity when fed a CA diet. In addition, activation of FXR has many other mechanisms to downregulate bile acid synthesis and govern the composition of the bile acid pool [21, 66–73]. Additionally, activation of FXR in the liver may also regulate and inhibit CYP8B1 in some ways [61].

**Figure 3:**
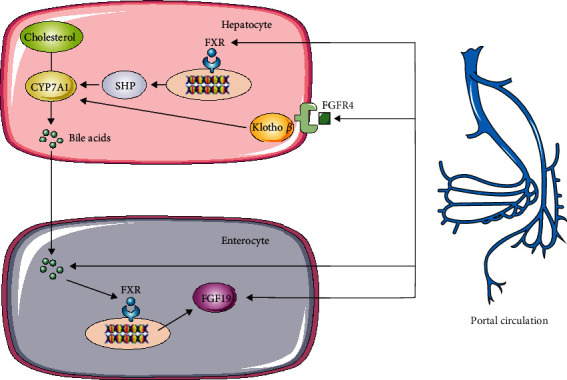
The relationship between total bile acids, FXR, and FGF19.

Numerous studies have shown that FXR is closely related to glucose metabolism. Diabetic rats induced by streptozotocin exhibited reduced FXR expression in the liver, which can be restored by insulin supplementation [74]. FXR knockout mice exhibited a distinct response to refeeding in hepatic expression of glucose metabolism genes [74]. Elevated glucose influx and activation of insulin signaling may lead to postprandial bile acid synthesis [75]. A previous study demonstrated that high concentrations of bile acids stimulate ligand-dependent FXR transactivating activity and increase cellular glucose flux [76]. During the postprandial phase, FXR may be activated to regulate glucose homeostasis [75, 77, 78]. Recent studies suggested that FXR regulates the sensitivity and secretion of peripheral insulin and promotes glycogen synthesis by inducing FGF15 (FGF19 in humans) in the intestine [70]. In addition, FGF19 directly activates SHP by combining with FGFR4 with the help of klotho *β* in hepatocytes [60] (Figure 3).

Though the concrete role of FXR in the regulation of hepatic glucose metabolism remains debatable, the probability that FXR activation inhibits hepatic glucose synthesis to decrease fasting plasma glucose has been universally acknowledged [79]. Its natural association with T2DM may decrease the quantity, activation, or sensitivity of FXR in some ways, and one study by Staels and Fonseca suggested that insulin suppresses expression of the FXR gene, speculating that diabetes may be associated with the dysbiosis of FXR expression [71–73].

### 4.2. Regulation of Glucose Homeostasis and Bile Acid Metabolism by TGR5

**Figure 4:**
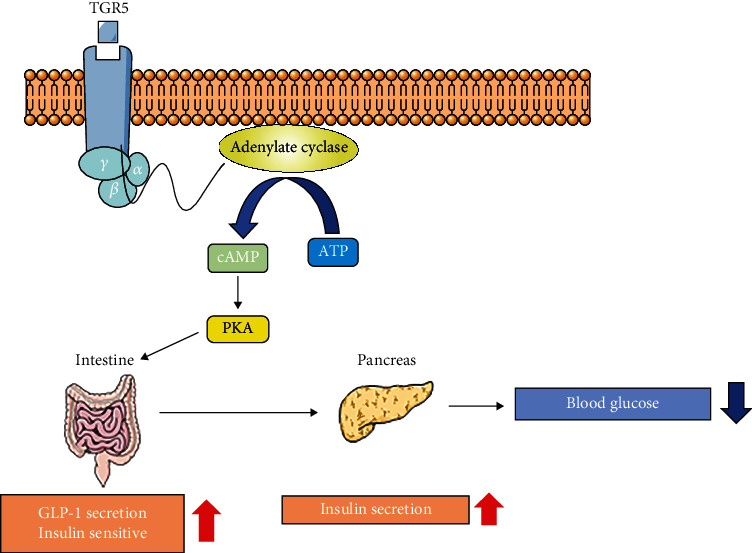
The relationship between TGR5, PKA, and GLP-1.

Transmembrane G protein-coupled receptor 5 (TGR5) is expressed in nearly the entire body, particularly in a variety of liver cells [61]. Multiple studies have shown that there is a tight connection between TGR5 and bile acid metabolism [79–81]. TGR5 might also regulate bile acid metabolism [82]. Distinct from FXR, the affinity of bile acids for hydrophobicity to TGR5 is LCA>DCA>CDCA>CA>UDCA [61]. Combined with its agonists, TGR5, with the help of its cofactors *α*, *β*, and *γ*, activates protein kinase A (PKA) signaling pathways by activating adenyl cyclase, leading to the swift growth of intracellular cAMP production [77]. Then, PKA pathways lead to the phosphorylation and induce the expression of the target genes of the transcription factor cAMP-responsive element-binding protein (CREB) [83] (Figure 4).

Glucose regulation improves when TGR5 signaling increases [81, 82]. Glucagon-like peptide 1 (GLP-1) promotes insulin secretion by islet beta cells in a glucose-dependent manner and reduces glucagon secretion by islet alpha cells, thereby lowering blood glucose. Secretion of GLP-1 is enhanced in response to TGR5 signaling activation in gastrointestinal enteroendocrine L cells [82]. In addition to its glucose-dependent insulinotropic effect, GLP-1 has similar properties to glucagon and induces satiety. Bile acids may regulate glucose homoeostasis, appetite, and even body weight *via* TGR5 [84–86]. Some studies have reported that TGR5 is a downstream target of FXR and it is required for promoting GLP-1 secretion through L cell FXR signaling [78]. The specific role of TGR5 in the regulation of glucose homeostasis requires further study, but activation of TGR5 does convey an apparent beneficial effect on glucose homeostasis.

### 4.3. Role of FXR, TGR5, and Total Bile Acids in Glucose Homeostasis and T2DM Remission after Bariatric Surgery

Although T2DM lacks a specific treatment, bariatric surgery, including Roux-en-Y gastric bypass (RYGB) and vertical sleeve gastrectomy (VSG), conveys long-term disease mitigation [87, 88]. Fibroblast growth factor 19 (FGF19), an intestinal feedback signal of bile acids, has been implicated in the glucometabolic changes that take place after Roux-en-Y gastric bypass (RYGB). Bariatric surgery improves glucose regulation by promoting bile acid signaling, which may then increase circulating bile acid concentrations or anatomical rearrangement of the gastrointestinal tract [89–91]. Studies have revealed that FXR and TGR5 contribute to the metabolic benefits of bariatric surgery. FXR is beneficial to weight loss and improves glucose regulation in response to VSG and TGR5 [89–91]. Improvements in signaling are associated with a TGR5-dependent pathway that decreases the hydrophobicity of the circulating bile acid pool [53]. This beneficial spectrum change in bile acids is associated with decreased expression of TGR 5-dependent liver CYP8B1 protein, with no effect on the expression of liver CYP7A1 [89].

A recent study investigating the relationship between total bile acids, FGF19, and T2DM in bariatric surgery identified important roles of total bile acids and FGF19 in T2DM remission after sleeve gastrectomy (SG) by comparing postprandial gut hormone patterns between patients undergoing laparoscopic gastric bypass (GB) and laparoscopic sleeve gastrectomy 2 years after surgery [92]. This research revealed that both laparoscopic GB and laparoscopic SG have significant effects after surgery but do have discrepancies not associated with insulin secretion, weight loss, or hindgut effect that are connected with reduced insulin resistance and duodenal exclusion in GB in T2DM remission. Moreover, differential regulation of different subtypes of ghrelin and total bile acids might be involved in the differing insulin resistance and T2DM remission responses between GB and SG procedures [92].

## 5. Bile Acid-Based Therapy for T2DM

### 5.1. Bile Acids as Therapeutic Drugs

Bile acids have been used for cholestatic liver diseases and metabolic diseases for years [93]. DCA, transformed by rectal taurocholate (TCA), increases GLP-1 secretion and insulin, leading to decreased serum glucose by activating intestinal bile acid receptors FXR and TGR5 [94, 95]. Although UDCA is not used for T2DM directly, it has been used to treat obese patients [96]. Short-term UDCA administration activates FXR to stimulate bile acid and cholesterol synthesis, while circulation of FGF19 decreases. Metformin is a noted drug prescribed for T2DM that improves insulin resistance and sensitivity. One of the drug's effects is increasing TUDCA and GUDCA by altering the gut microbiota. In addition, TUDCA and GUDCA act as antagonists of intestinal FXR to improve hyperglycemia in T2DM [72]. Therefore, another way to treat T2DM may be to increase concentrations of TUDCA and GUDCA directly.

### 5.2. Bile Acid Sequestrants

Bile acid sequestrants were observed to improve glycemic control in T2DM patients as early as the 1990s [31]. Since bile acids have been proven to play an important role in glucose metabolism, which is relevant to T2DM and insulin resistance, bile acid sequestrants were explored.

First used as a treatment for hypercholesterolemia, bile acid sequestrants, such as cholestyramine, colesevelam, colestimide, and colestipol, are nonabsorbable resins that combine negatively charged bile salts into a complex in the intestinal lumen, which are then excreted in the feces; therefore, bile acids are diverted from the enterohepatic cycle and excreted from the body [97]. Bile acid sequestrants decrease circulating concentrations of LDL cholesterol, the substrate for bile acid production, *via* increasingly delivering LDL cholesterol to the liver and enhancing cholesterol synthesis and upregulation of LDL receptors [31]. Cholestyramine functions as a combination of bile acids and is a therapeutic option for some metabolic syndromes, such as dyslipidemia [98]. It stimulates cholesterol transformation into bile acids by decreasing the concentration of bile acids returned to the liver *via* enterohepatic cycling.

Recently, bile acid sequestrants have been approved in the USA for the treatment of T2DM, despite the mechanisms of action still not being completely understood [99]. Bile acid sequestrants successfully treat T2DM due to their hypoglycemic effect. Bile acid sequestrants could alter the bile acid pool composition. However, this hypothesis was not supported by clinical research findings, which suggested that alterations of bile acid pool composition are not a significant pathway in the glucose-lowering action of bile acid sequestrants [61]. In 2008, colesevelam was approved by the FDA for the treatment of T2DM [28, 29]. Two weeks of colesevelam treatment in T2DM patients altered the synthesis of specific bile acids, which affected the concrete composition of the total pool size [42]. However, animal studies indicated that colesevelam improved oral glucose tolerance by activating TGR5 on L cells with subsequent GLP-1 secretion. Although the hypothesis that BAs may activate TGR5 to modulate human intestinal GLP-1 release and glucose homoeostasis remains to be further understood, the TGR5 signaling pathway may represent a target that may provide a highly promising strategy for the treatment of T2DM.

### 5.3. Farnesoid X Receptor Agonists

Currently, studies of FXR-targeting therapies for T2DM are extremely limited. Obeticholic acid (OCA), a semisynthetic bile acid, is 30 times more effective in activating FXR than CDCA. Although it was not used for diabetes treatment, its effects in inhibiting bile acid synthesis and improving liver function have been verified in the treatment of metabolic liver diseases [100, 101]. Since FXR activation inhibits hepatic glucose synthesis to decrease fasting plasma glucose, it is likely that similar to OCA, targeting FXR represents a novel strategy for improving hyperglycemia in T2DM.

### 5.4. G Protein-Coupled Bile Acid Receptor Agonists

**Figure 5:**
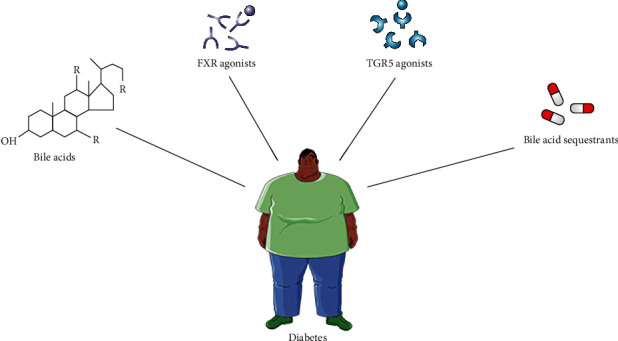
Bile acid-based therapy for T2DM.

Intestine-selective TGR5 agonists may represent a potential strategy for T2DM therapy, as they have been observed to improve glucose homeostasis [21]. Insulin resistance is a characteristic of T2DM, although the mechanism of its occurrence has yet to be elucidated, but there are two primary theories. First, lipid overload, in which fat cell enlargement leads to increased levels of circulating free fatty acids (FFAs) and its metabolites, as well as deposition in nonfat cells, inhibits insulin signaling. Second, the inflammation doctrine postulates that enlarged fat cells attract macrophages, which secrete inflammatory signaling molecules, such as TNF-*α*, resistin, and IL-6 [102]. These two theories intersect and complement each other. INT-777, a semisynthetic acid that activates TGR5, can decrease lipid loading and macrophage inflammation by inhibiting nuclear factor *κ*B (NF-*κ*B) and production of proinflammatory cytokines (Figure 5).

## 6. Conclusions

In this review, we focused on the role of bile acids in glucose metabolism and the occurrence and development of T2DM, as well as the possibility of bile acids representing a new target for treating T2DM. Without a doubt, bile acids are exceedingly significant in glucose metabolism. Their important roles in the occurrence and development of T2DM have drawn plentiful attention. A mass of research findings have shown that there might be transformations of bile acids in their metabolism concomitant with the occurrence of T2DM, to some extent, leading to abnormal glucose metabolism and insulin resistance. However, additional studies are needed to verify these alterations and to understand the specific interactions that are occurring. Additionally, further animal experiments and clinical trials are required to support the safety and efficacy of bile acids and their sequestrants. Importantly, novel methods of targeted therapy for T2DM have recently been identified, and the future of T2DM treatment is becoming more promising.

## Figures and Tables

**Table 1:** The differential affinity of the subtypes of bile acids towards FXR and TGR5.

Subtypes of bile acids	
FXR agonists	6-ECDCA^43^ (synthetic)>CDCA^44,45,46^>CA>DCA>LCA
FXR antagonists	T*α*MCA, T*β*MCA^47^
TGR5 agonists	INT-777^48^ (a derivative of CDCA)>LCA^49,50^>DCA^51^>CDCA>CA>UDCA
TGR5 antagonists	SBI-115^52^ (synthetic)
